# Pb103 Regulates Zygote/Ookinete Development in *Plasmodium berghei* via Double Zinc Finger Domains

**DOI:** 10.3390/pathogens10121536

**Published:** 2021-11-24

**Authors:** Makoto Hirai, Akimasa Maeta, Toshiyuki Mori, Toshihiro Mita

**Affiliations:** Department of Tropical Medicine and Parasitology, Faculty of Medicine, Juntendo University, 2-1-1 Hongo Bunkyo-ku, Tokyo 113-8421, Japan; aaaa1125bbbb@gmail.com (A.M.); moritoshi@biken.osaka-u.ac.jp (T.M.); tmita@juntendo.ac.jp (T.M.)

**Keywords:** *Plasmodium berghei*, RNA binding protein, zinc finger, translational repression

## Abstract

Sexual reproduction of *Plasmodium* parasites takes place in anopheline mosquitoes, where male and female gametes fuse to form zygotes and then ookinetes. These processes are orchestrated by stage-specific protein expression, which is mediated in part by translational repression. Accumulating evidence shows that RNA binding proteins (RBPs) play crucial roles in these processes. Here, we report the characterization of *P. berghei* 103 (Pb103), which encodes a protein possessing double zinc finger domains (ZFs), an RBP. Reporter parasites expressing azami green fluorescent protein (AGFP) under the endogenous *Pb103* gene promoter (*Pb103-AGFP* reporter) showed that the AGFP fluorescent signal was detected from gametes to ookinetes, while *AGFP* mRNA was translationally repressed in female gametocytes. The *Pb103*-disrupted parasites (*Pb103(−)*) grew and produced gametocytes with similar efficiencies to those of wild-type parasites. However, no oocysts were formed in mosquitoes fed *Pb103(−)*. An in vitro fertilization assay showed abortion at the zygote stage in *Pb103(−)*, suggesting that Pb103 plays a critical role in zygote/ookinete development. Cross-fertilization assays with *Pb103(−)* and male- or female-sterile parasites revealed that Pb103 was essential exclusively for female gametes. To identify the domains critical for zygote/ookinete development, transgenic parasites expressing partially deleted Pb103 were generated and assayed for ookinete maturation. As a result, deleting either of two ZFs but not the C-terminal region abolished zygote/ookinete development, highlighting the indispensable roles of ZFs in parasite sexual development, most likely via translational repression.

## 1. Introduction

Malaria is one of the most life-threatening parasitic diseases. There are 229 million clinical cases and 409,000 deaths, and the main victims are children under 5 years old living in sub-Saharan Africa [1]. The causative agents of malaria are *Plasmodium* spp., which are transmitted by anopheline mosquitoes. In vertebrate hosts, a subpopulation of asexually proliferating malaria parasites differentiates into male and female gametocytes. Once gametocytes enter the mosquito midgut by blood feeding, they develop into mature gametes in 8 to 15 minutes. The male gametes undergo three rounds of DNA replication and release eight flagella through a process called exflagellation [2,3]. Each male flagellum fertilizes a female gamete via membrane fusion factors, such as HAP2/GCS1 [4,5,6], in one hour. After fertilization, the zygote is converted into a motile ookinete in the next 16–18 h [7]. Uncovering the molecular mechanisms behind these developmental processes would provide a clue for the identification of transmission-blocking vaccine molecules that target parasite development in mosquitoes [8,9].

Recent accumulating evidence shows that the parasite’s life cycle is tightly coordinated by stage-specific transcription and translation. For transcriptional regulation, apicomplexan Apetala-2 (Api-AP2) transcription factors [10] regulate the expression of the genes essential for each developmental stage. AP2-G determines sexual commitment (gametocytogenesis) [11,12]. In female gametocytes, AP2-G2 represses asexual gene expression [11,13], while AP2-FG activates female-specific genes [14], whereby gametogenesis and zygote development proceed. Subsequent ookinete development is regulated by the AP2-O family [15,16]. Apart from transcriptional regulation, translational regulation also plays a crucial role in the parasite life cycle, especially at the moment of host switching. It has been reported that female gametocytes store mRNAs, such as *P25*, *P28*, *CCP2*, and *AP2-O*. These mRNAs are translationally silent to preadapt to drastic environmental changes incurred by introducing gametocytes into the mosquito body [17]. It has been reported that the DDX6-class DEAD-box RNA-helicase DOZI and its interacting partner, CITH (CAR-I/Trailer Hitch Homolog) of *P.* *berghei*, play a central role in the translational repression of the above-mentioned mRNAs at gametocyte stages [18,19]. DOZI colocalizes with CITH as messenger ribonucleotide proteins (mRNPs) in stress granules, where transcripts are stored in the quiescent state for translation for a later time [18]. Deletion mutants of DOZI and CITH can fertilize and differentiate to zygote but fail in ookinete development [19]. DOZI and CITH deletion mutants showed that the expression level of 370 or 183 transcripts, respectively, dropped to less than half because of the instability of these transcripts in the absence of DOZI and CITH [18,19]. These demonstrate that the fine tuning of DOZI/CITH-mediated translation is critical for ookinete development. In eukaryotes, RNA-binding proteins (RBPs) play crucial roles in post-transcriptional regulation, such as translational repression, RNA transport, and degradation. In particular, it has been reported that RBPs repress the translation of maternal mRNAs, which are necessary for early gamete development immediately after fertilization [20]. The importance of RBPs for cell development and differentiation is corroborated by the high degree of conservation of RBPs in a wide variety of eukaryotes from yeast to humans [21] as well as Plasmodium [22,23]. Among a dozen RNA binding proteins, RNA helicases, zinc-finger domains (ZFs), K homology (KH), Pumilio and Fem-3 binding factor (Puf), RNA recognition motifs, and acetylation lowers binding affinity (Alba) families have been reported in *Plasmodium* [18,24,25,26,27]. To understand RBP-mediated translational repression in gametocytes, we searched for genes coding for RBPs whose expression is predominant in gametocytes. Among several candidate genes, we report the characterization of Pb103-containing double ZF domains. Employing a gene targeting strategy, the function of Pb103 on the parasite life cycle was analyzed with an emphasis on detailed structural characteristics by using partially deleted Pb103 mutants.

## 2. Results and Discussion

### 2.1. Pb103 Is Conserved in Plasmodium

**Figure 1:**
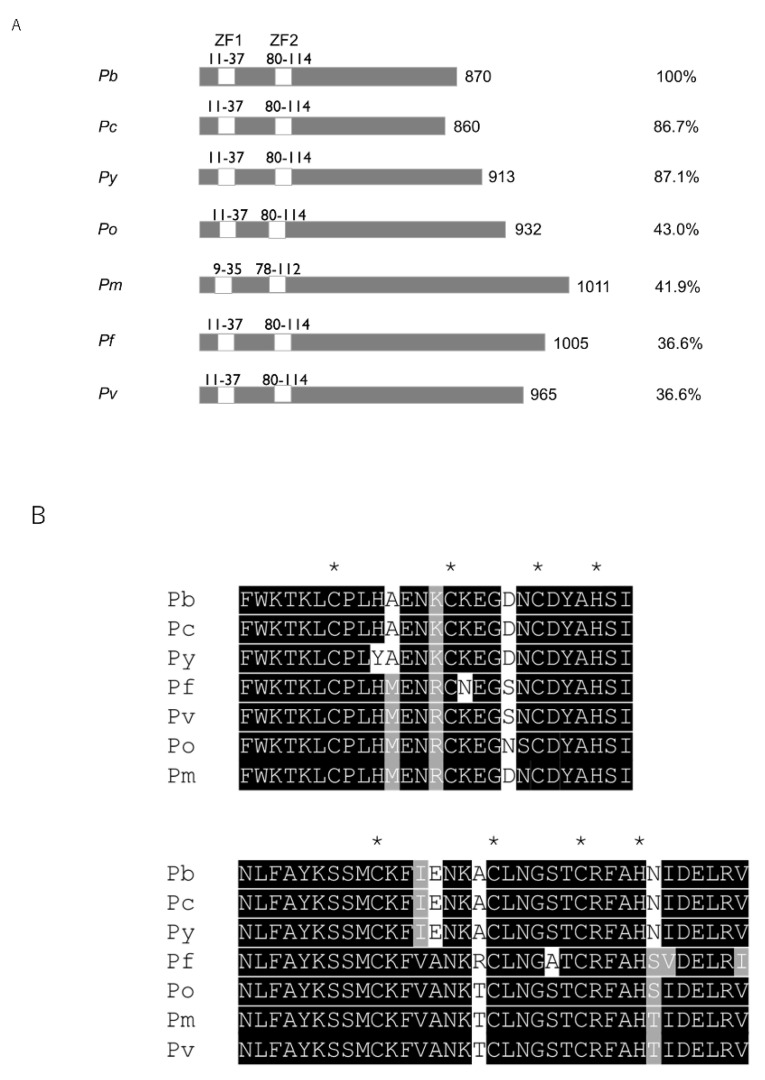
Comparison of the structure of Pb103 and orthologs. (**A**) Primary structure of Pb103 orthologs in malaria parasites. White boxes and numbers indicate the position of the first and the second zinc finger domains. The right number indicates the amino acid length. The right-most number is the identity of each ortholog to Pb103. Gene accession numbers are as follows: *Pb* (*P. berghei*), PBANKA_1134900; *Pc* (*P. chabaudi*), PCHAS_1134400; *Py* (*P. yoelii*), PY17X_1136400; *Po* (*P. ovale*), PocGH01_11021900; *Pm* (*P. malariae*), PmUG01_11027400; *Pf* (*P. falciparum*), PF3D7_1358600; *Pv* (*P. vivax*), PVP01_11133009. (**B**) Comparison of the amino acid sequences of 1st (upper) and 2nd (lower) zinc finger domains among Pb103 orthologs. Identical and conserved residues are shaded black and gray, respectively. CCCH motif is indicated by asterisks.

We searched PlasmoDB for RBP-coding genes whose expression is high in gametocytes to identify novel translation regulators functioning in parasite reproduction stages. Among several candidate genes, we focused on PBANKA_1134900, which encodes 870 amino acids with a molecular weight of approximately 103 kDa, and designated it Pb103. InterProScan analysis revealed that Pb103 possesses two CCCH-type ZF domains. A database search showed that Pb103 is highly conserved among rodent malaria parasites (Figure 1A). While orthologous genes are also detected in human malaria parasites with low identity at the entire sequence level, two ZF domains are highly conserved among rodent and human malaria parasites (Figure 1B). All these genes contain a single exon, which is another conserved feature in these orthologous genes. The presence of a Pb103 ortholog in various *Plasmodium* species suggests that the Pb103 ortholog may have conserved roles in parasite development.

### 2.2. Expression Analysis of Pb103 by Using the Pb103-AGFP Reporter

**Figure 2:**
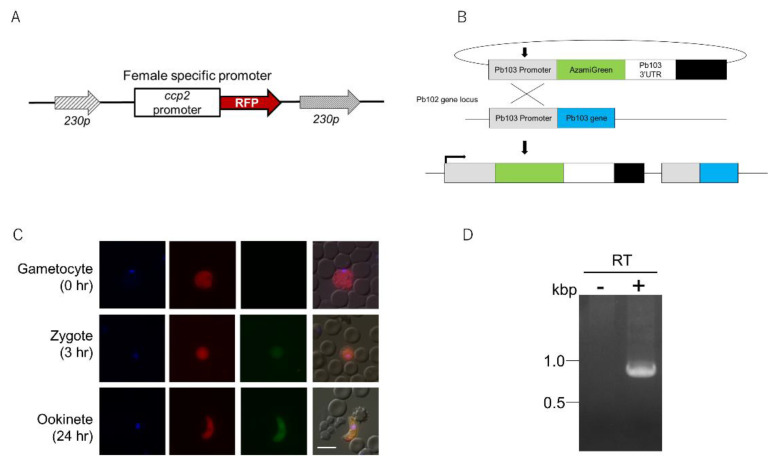
Generation of reporters. (**A**) Generation of *female RFP* reporter, which is a recipient parasite. Rough- and fine-dashed arrows indicate 5′ and 3′ UTR of the *230p* gene, respectively. The red arrow indicates the *RFP* gene. The white box indicates the *ccp2* gene promoter, which is exclusively active throughout female gametocyte to ookinete stages. This DNA fragment was integrated into the *230p* gene locus by double-crossover homologous recombination. (**B**) Schematic representation of *Pb103-AGFP* reporter generation using a plasmid integration through single crossover homologous recombination. The boxes indicate the *Pb103* promoter (gray), *AGFP* (green), 3′ UTR of the *Pb103* gene (white), and a selectable marker (black). For the transfection, the plasmid was linearized by *PacI* site located in *Pb103* promoter and introduced in a *female RFP* reporter, as shown in (**A**). (**C**) Expression analysis of the *Pb103-AGFP* reporter. The *female RFP* reporter was transfected with the *Pb103-AGFP* plasmid, as described in (**B**). The resulting *Pb103-AGFP* reporter was subjected to in vitro fertilization assay. The fluorescent signal was observed at 0 (top), 3 (middle), and 24 h (bottom) post-gametogenesis. The nucleus was stained with DAPI (1st column). RFP (2nd column), AGFP (3rd column), and merged images (last column) were shown. The white bar represents 5 μm. (**D**) Detection of *AGFP* mRNA by RT-PCR. RNA was prepared from gametocyte-rich fraction and treated with DNase-I. A reverse transcribed (+) sample was used as a template for PCR. RNA without reverse transcription was used as negative control (−).

According to RNA-seq data [28], the *Pb103* gene is exclusively expressed in female gametocytes. To confirm this, we attempted to generate a *P. berghei* reporter line expressing AGFP fused to the C-terminal of the Pb103 protein under the endogenous *Pb103* promoter. The resulting transgenic parasite did not show any AGFP signal during the whole life cycle (data not shown). This could be due to the steric hindrance of AGFP caused by Pb103 fusion or proteolytic processing. Then, we changed our strategy to a generation of another transgenic line expressing AGFP driven by the endogenous *Pb103* promoter. To detect female gametocytes easily, we first generated a selection marker-free *female RFP* reporter that expresses RFP throughout female gametocytes to ookinete under the control of the *ccp2* promoter, which is known to be active in those developmental stages [29] (Figure 2A). Next, we transformed the *female* *RFP* reporter with the Pb103-AGFP plasmid (Figure 2B), whereby AGFP expression in RFP-positive cells could be monitored easily. Tail blood was taken from mice infected with the *Pb103-AGFP* reporter, and the fluorescent signal was observed. Unexpectedly, no AGFP signal was detected in RFP-positive or RFP-negative cells (Figure 2C, upper panels). This suggests that *AGFP* is not transcribed or *AGFP* mRNA is not translated. To investigate whether the *AGFP* gene is transcribed, we performed RT-PCR using RNA isolated from gametocyte-rich cell fractions. As a result, *AGFP* mRNA was detected (Figure 2D), indicating that *AGFP* mRNA is in a translationally quiescent state. Next, the same infected blood was incubated in a gametogenesis-inducing medium. The AGFP signal was exclusively detected in RFP-positive cells at 3 and 24 h postinduction (Figure 2C, middle and bottom panels), demonstrating that AGFP is translated in zygotes and ookinetes. Considering the translational repression of *AGFP* mRNA, the results suggest that *Pb103* mRNA translation could also be repressed and then initiated after gametogenesis. Such stage-specific translational regulation suggests that Pb103 may have crucial roles in parasite development after gametogenesis.

### 2.3. Pb103 Is Essential for Parasite Reproduction

**Figure 3:**
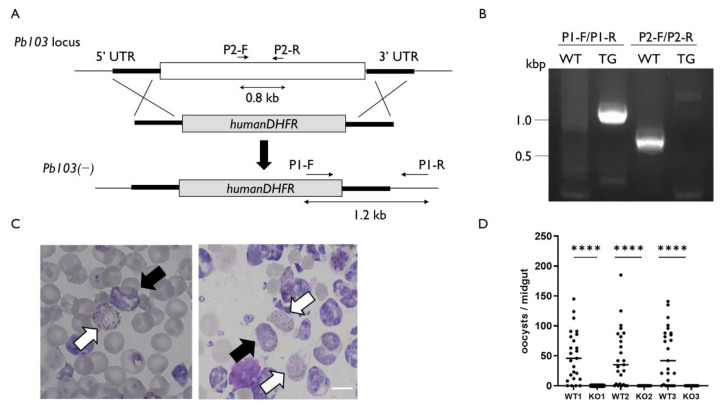
Targeted disruption of the *Pb103* gene and phenotypes of *Pb103(−)*. (**A**) Schematic representation of replacement strategy to generate *Pb103(−)*. The coding region of the *Pb103* gene is replaced with *human* DHFR, which is a selectable marker by double-crossover homologous recombination through the 5′ and 3′ UTR of the *Pb103* gene. The directional and double arrows indicate the primer position and the expected product size, as shown in (**B**). (**B**) Diagnostic PCR. Genomic DNA of *Pb103(−)* (TG) and wild-type (WT) were used as templates. P1-F/P1-R and P2-F/P2-R are specific to TG and WT, respectively. The expected size is 1.2 kb for TG and 0.8 kb for WT. (**C**) Thin blood smear prepared from mice infected with wild-type (left) or *Pb103(−)* parasites (right). The slides were stained with Giemsa. Black and white arrows indicate female and male gametocytes, respectively. The bar represents 5 μm. (**D**) Infectivity of *Pb103(−)* (KO) and wild-type (WT) to mosquitoes. The oocyst numbers per mosquito carrying KO or WT are plotted. The results of three independent experiments are shown. Horizontal bars indicate the median. Stars represent a statistically significant difference between WT and KO in each experiment (Mann–Whitney test: **** *p* < 0.0001).

**Figure 4:**
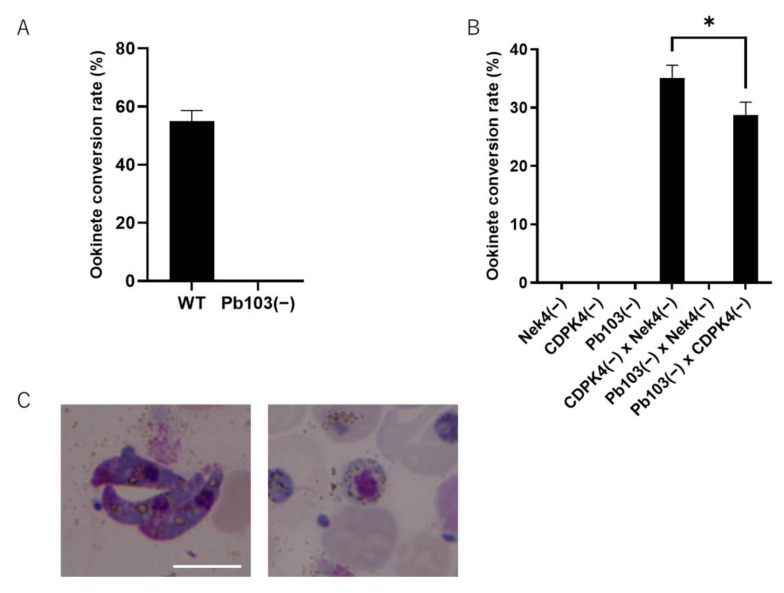
In vitro fertilization assay of *Pb103(−)* and wild type. (**A**) The ratio of females that successfully fertilized males and differentiated into mature ookinetes is expressed as ookinete formation rate. The bar represents mean ± SD (*n* = 3). (**B**) Giemsa staining of the samples after in vitro fertilization assay of *Pb103(−)* (right) and wild type (left). The mature ookinete (left) and zygote (right) are shown. The bar represents 5 μm. (**C**) In vitro fertilization assay. Self-fertilization of *CDPK4(−)* (female fertile), *Nek4(−)* (male fertile), and *Pb103(−)*, and cross-fertilization between two lines among those three parasites was performed. The ookinete formation rate is shown. Data are from three independent experiments. (*t*-test: ∗ *p* < 0.02).

To address the functions of Pb103, we generated *Pb103(−)* parasites. The disrupting construct, which contains the selectable marker *human DHFR*, targeted 500 bp upstream and downstream of the *Pb103*-coding region (Figure 3A, upper and middle panels). *Pb103* gene deletion was conducted by double-crossover homologous recombination, whereby the *Pb10*3 gene was replaced by the selectable marker (Figure 3A, lower panel). The correct gene replacement in the *Pb103(−)* clone was confirmed by diagnostic PCR (Figure 3B). The morphology of *Pb103(−)* gametocytes was indistinguishable from that of wild-type parasites (Figure 3C). *Pb03(−)* differentiated into gametocytes with similar efficiency as wild type (Table 1). We investigated the infectivity of *Pb103(−)* in mosquitoes. Mosquitoes were fed on mice infected with either *Pb103(−)* or wild type and were then dissected for the evaluation of parasite development on day 16 post feeding. Three independent experiments showed that oocysts in the midgut were detected in mosquitoes fed on mice carrying wild-type parasites. In contrast, no oocysts were detected in the mosquitoes fed on mice carrying *Pb103(−)* (Figure 3D). This result indicates that Pb103 is required for parasite development in mosquitoes. To ensure that no infectious *Pb103(−)* sporozoites existed in the salivary glands, we prepared salivary gland extracts from mosquitoes fed *Pb103(−)* or wild type and injected them into mice intravenously. As a result, the mice injected with the extracts from four to six mosquitoes carrying wild-type oocysts were infected, while no infection was detected in mice injected with the extract prepared from 15 and 20 mosquitoes fed with *Pb103(−)* (Table 2). Since *Pb103(−)* could not develop into oocysts and no infectious sporozoites were produced, it is likely that *Pb103(−)* parasites may halt development before oocyst stages. To investigate which steps of the developmental process are defective in *Pb103(−)*, we performed an in vitro fertilization assay that mimics the gametogenesis and fertilization taking place in the mosquito midgut [30]. In the wild type, 54.3% of female gametes were fertilized with male gametes and then transformed into ookinetes (Figure 4A,B, left panel). In *Pb103(−)*, on the other hand, no mature ookinetes but zygotes were detected (Figure 4A,B, right panel). These results suggest that Pb103 has critical roles after zygote development.

As shown in the protein expression analysis of the *Pb103-AGFP* reporter, Pb103 is translated after gametogenesis in females. Therefore, the developmental failure observed in *Pb103(−)* parasites could be exclusively attributed to females. To investigate this possibility, we performed in vitro cross-fertilization experiments between *Pb103(−)* and *CDPK4(−)* (male infertility) or *Nek4(−)* (female infertility) gametes. The self-fertilization of *CDPK4(−)* and *Nek4(−)* did not produce ookinetes, while *CDPK4(−)* × *Nek4(−)* cross produced ookinetes, which was equivalent to 35.1% of *CDPK4(−)* female gametes fertilized to *Nek4(−)* males (Figure 4C). Cross-fertilization of *Pb03(−)* × *CDPK4(−)* produced ookinetes with slightly lower efficiency compared to *CDPK4(−)* × *Nek4(−)* crosses (*p* < 0.01; Figure 4C). On the other hand, the *Pb03(−)* × *Nek4(−)* cross failed to generate mature ookinetes. These results demonstrate that Pb103 is exclusively required in females.

### 2.4. Zinc Finger Domains Are Indispensable for Ookinete Maturation

**Figure 5:**
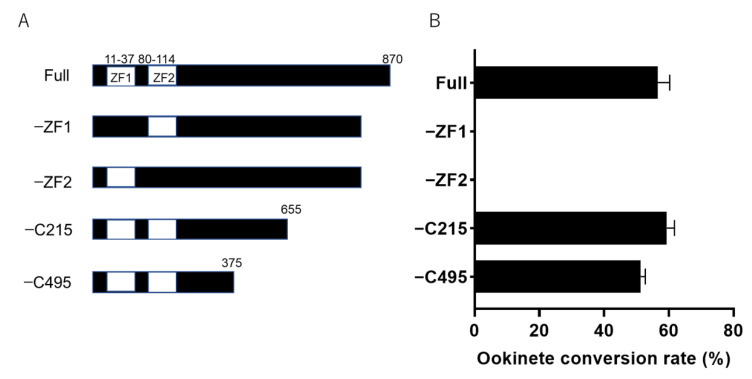
Characterization of Pb103 domains essential for ookinete maturation. (**A**) Grammatic representation of the partially deleted Pb103 products. The top bar is the normal Pb103 protein (Full). The −ZF1 and −ZF2 lack respective domains. −C215 and −C495 indicate that the respective numbers of amino acids are deleted from the C-terminal. The position of ZF1 and ZF2 are indicated by white boxes. (**B**) In vitro fertilization assay of four transgenic and wild-type parasites. Error bars represent mean ± SD (*n* = 3).

To identify crucial domains for ookinete maturation, we made four Pb103 deletion mutants and designated them −ZF1, −ZF2, −C215, and −C495 (Figure 5A). The −ZF-1 or −ZF-2 lacks the first or second ZF domain, while −C215 or −C495 lacks the respective numbers of amino acids from the C-terminus. These mutants were generated by genome edition, and the desired deletions in all clones were confirmed by Sanger sequencing. These mutants were subjected to an in vitro fertilization assay to investigate the functional domain based on the morphological maturation of ookinetes. As a result, obvious mature ookinetes were frequently detected in −C495 and −C215 with similar efficiency to that of wild-type (full) parasites (Figure 5B). Notably, mature ookinetes were generated in −C495, which lost more than half of the full length from the C-terminus. As we mentioned in the construction of the *Pb103-AGFP* reporter, the reporter parasites expressing the Pb103 protein fused to AGFP did not show a fluorescent signal. Considering this and the successful ookinete maturation of the −C495 mutant together, it is conceivable that Pb103 may be cleaved in the C-terminus. This could be the reason for the lack of signal detection in the Pb103-AGFP-expressing parasites. In addition, the −ZF1 and −ZF2 parasites did not produce mature ookinetes. These results highlight the importance of the ZF domains in Pb103 for ookinete maturation.

### 2.5. Mechanisms of Post-Translation Repression of Pb103

By using a *Pb103-AGFP* reporter, we showed that *AGFP* mRNA was translationally repressed in gametocytes and that this repression was released by gametogenesis. In the *Pb103-AGFP* reporter, the *AGFP* gene is under the control of the 5′ and 3′ UTRs of the *Pb103* gene, suggesting that translational regulatory elements, if any, would exist in these UTRs. It has been reported that a U-rich element in the 5′ or 3′ UTR is essential for the translational repression of *P28* and *P25* in female gametocytes [31,32]. Several U-rich regions are detected in the 5′ and 3′ UTRs of *Pb103*. However, the genome of *Plasmodium* is extremely AT-rich (www.plasmodb.org, accessed on 12 December 2016). Thus, it is difficult to identify such elements based on U-richness. Further study is needed for functional identification of the elements to which regulatory factors get access to halt the translation of *Pb103* mRNA. It has been reported that the DOZI/CITH RNA helicase is a central factor in translational repression in gametocytes [18]. The DOZI/CITH complex is a component of mRNPs, which prevents target mRNAs from translation and degradation. RNA immunoprecipitation assays and microarrays revealed that 731 mRNAs were associated with DOZI/CITH, and half of them were translated into ookinetes. The functions of these proteins are speculated to be involved in ookinete maturation, such as motility, adhesion, and cell traversal [33]. In addition, both DOZI and CITH deletion mutants could fertilize and differentiate into zygotes but failed in ookinete formation [19]. Importantly, the RNA immunoprecipitation assay data showed that *Pb103* mRNA was bound by DOZI/CITH, and the level of *Pb103* mRNA was a 6.57-fold reduction in the DOZI mutant [32]. These results suggest that the stability and translation of *Pb103* mRNA could be under the control of DOZI/CITH. Among 731 mRNAs of potential targets for DOZI/CITH, only a single gene deletion, *Pb103* deletion, showed a severe defect in ookinete maturation, suggesting the functional importance of Pb103 for DOZI/CITH-mediated ookinete maturation. In the current study, our main effort was made to elucidate the structural importance of the Pb103 protein by making four Pb103 deletion mutants and their phenotypic study. Our data revealed that over half of the C-terminal region was not necessary for ookinete maturation. In contrast, we found that either of the ZF domains was essential for ookinete maturation. It is plausible that double ZF domains of Pb103 function as RNA binding to regulate the translation of target mRNAs. A previous study revealed that 15 RBP-coding mRNAs in addition to Pb103 are bound to the DOZI/CITH complex [32]. It is interesting to understand the interrelationship among these RBPs as well as other putative targets of DOZI/CITH to uncover the DOZI/CITH-mediated translational regulation cascade. In this context, for further work, the identification of target mRNAs for Pb103 is of special interest because this would provide clues for an in-depth understanding of the molecular machinery of DOZI/CITH and Pb103-mediated posttranscriptional regulation. In addition, this study may also help to identify new candidate molecules of transmission-blocking vaccine strategies that target parasite development in mosquitoes.

## 3. Methods

### 3.1. Ethics Statement

The protocols for all animal and recombinant DNA experiments were approved by the Ethics of Experimental Animals Committee and Recombinant DNA Committee of School of Medicine, Juntendo University, and the assigned numbers were no. 2021036 and no. 25-115, respectively.

### 3.2. Animals and Parasites

BALB/c mice (female, 5 weeks old) were purchased from Japan SLC, Inc. and were used throughout the experiments. The rodent malaria parasite *Plasmodium berghei* ANKA (clone 2.34) was used for transgenic parasite generation. *Anopheles stephensi* (SDA 500 strain) was reared under a photoperiod of 14:10 h (light:dark) at 26 °C.

### 3.3. Generation and Expression Analysis of the Pb103-AGFP Reporter

The pL1186 plasmid was previously designed to make a transgenic line that expresses azami green (AGFP) and red fluorescent proteins (RFPs) for male and female gametocytes to ookinete, respectively [29]. The pL1186 plasmid was digested with *EcoRV*/*NotI* and self-ligated to remove the male-specific promoter and AGFP. The resulting plasmid (female-RFP) was digested with two *SacII* sites and introduced into the *230p* gene locus by double-crossover homologous recombination by a previously reported protocol [34]. Subsequently, the transgenic parasite clones were subjected to negative selection using 5-fluorocytosine to remove the selectable marker gene. The resulting parasite clone (*female-RFP* reporter) expresses RFP throughout female gametocytes to ookinetes [35] and was used as a recipient parasite of the Pb103-AGFP plasmid described below. For the construction of the Pb103-AGFP plasmid, fragments covering the 5′ untranslated region (UTR) (−1.5 kb) and 3′ UTR (−1.5 kb) of *Pb103* and AGFP were amplified using Pb103-5′ UTR-F1/Pb103-5′ UTR-R1, Pb103-3′ UTR-F1/Pb103-3′ UTR-R1, and AGFP-F/AGFP-R. These three fragments were serially cloned in the order of Pb103-5′ UTR, AGFP, and Pb103-3′ UTR into *HindIII*/*PstI*-digested pL0006 by an In-Fusion HD cloning kit (TaKaRa). The final plasmid, Pb103-AGFP, was linearized by the *PacI* site in the 5′ UTR of *Pb103* and integrated into the recipient parasites by a single crossover. The final transformant, the *Pb103-AGFP* reporter, was selected by pyrimethamine. The fluorescent signal was monitored in asexual and gametocyte stages. To monitor the signal after gametocyte stages, gametogenesis and fertilization of the reporter were induced by a previously described protocol [4,30]. The fluorescent signal was observed at 0, 3, and 24 h postinduction under a fluorescence microscope (AxioImager M2, Zeiss, Tokyo, Japan).

### 3.4. Reverse Transcription PCR (RT-PCR)

The mice were intraperitoneally injected with 100 μL of phenylhydrazine hydrochloride (2.5 mg) on day 0 and were then injected with the freezing stock of wild-type parasites on day 2. The mice were fed water containing sulfadiazine (15 mg/L) on days 5 and 6 to kill asexual stage parasites. On day 7, whole blood (1 mL) was collected by cardiac puncture and mixed with 5 mL of lysis buffer (150 mM ammonium chloride, 10 mM potassium carbonate, and 1 mM EDTA) on ice to induce hemolysis. The blood was then centrifuged at 600× *g* for 6 min and washed with phosphate-buffered saline. Total RNA was extracted from this gametocyte-rich cell fraction and then treated with DNase-I using the FastGene™ RNA Premium Kit (NIPPON Genetics Co., Ltd., Tokyo, Japan). Total RNA (1 μg) was reverse-transcribed using the PrimeScript™ RT–PCR Kit (TAKARA BIO Inc., Shiga, Japan) and was used as a template for PCR to amplify the full-length *AGFP* gene using a pair of primers (AGFP-F/AGFP-R). RNA without reverse transcription was used as a negative control.

### 3.5. Targeting Disruption of Pb103, PbCDPK4, and PbNek-4 Genes

To generate a construct for *Pb103* gene disruption, two PCR fragments covering 500 bp of the 5′ and 3′ UTRs of the *Pb103* gene were amplified by two pairs of primers, Pb03(−)-F1/Pb03(−)-R1 and Pb03(−)-F2/Pb03(−)-R2, respectively, and *P. berghei* genomic DNA as a template. Then, each PCR product was conjugated to either end of *PstI*/*HindIII* digested-pL0006 (MRA-775; BEI Resources.) by fusion PCR using primer Pb03(−)-F1/Pb03(−)-R2 [36]. Similarly, knockout constructs for the *CDPK4* [37] and *Nek-4* [38] genes, which are essential for male and female fertility, were generated as follows. The 5′ and 3′ UTRs of *CDPK4* and *Nek-4* were amplified using CDPK4(−)-F1, -R1, -F2, and -R2 or Nek4(−)-F1, -R1, -F2, and -R2. Each of these fragments was fused to either the 5′ or 3′ end of *PstI/HindIII*-digested pL0006 using the primers CDPK4(−)-F1/CDPK4(−)-R2 and Nek4(−)-F1/Nek4(−)-R2. The final constructs were introduced to *P. berghei*, and the transformant was cloned.

### 3.6. Development of Pb03(−) in Mosquitoes and Transmission to Mice

A phenotypic study of *Pb03(−)* in mosquitoes was performed by following a previously described procedure [39]. In brief, female mosquitoes (4–7 days old) were fed on mice carrying either *Pb03(−)* or wild type, and fully blood-fed mosquitoes were collected. Sixteen days after feeding, the mosquitoes were dissected, and the numbers of oocysts in the midgut were counted. Mosquito feeding and subsequent cultivation were performed at 21 °C. To test parasite transmission from mosquitoes to mice, salivary glands from mosquitoes carrying *Pb03(−)* and wild-type parasites were collected in 100 μL of phosphate-buffered saline on ice. Salivary gland extracts (100 μL) were injected into each mouse. On day 5 after the injection, the mice were checked for infection by observing Giemsa-stained thin blood film smear slides. For the mice that were negative for infection, a blood smear test was conducted on day 16. The slides were observed under a microscope (×1000).

### 3.7. In Vitro Cross-Fertilization Assay

Fertilization among *Pb03(−)*, *CDPK4(−)*, *Nek4(−)*, or wild type was performed by an in vitro cross-fertilization assay, as described previously [4]. A pair of 5 μL of tail blood infected with either *Pb03(−)*, *CDPK4(−), Nek4(−),* or wild type was added immediately to 1 mL of gametogenesis-inducing medium (10% fetal bovine serum in RPMI1640, pH 8.2) at 21 °C for 12 min to induce gametogenesis and check for exflagellation. This mixture was further incubated for 24 h to induce fertilization and ookinete formation. The ookinete conversion rate was determined by the percentage of female gametocytes fertilized with males and transformed into a mature ookinete. The female gametocytes and ookinetes were stained with Giemsa and counted under a microscope (×1000).

### 3.8. Generation of Partially Truncated Pb103 Mutants

Four Pb103 partial deletion mutants were generated by using CRISPR/Cas9. For the construction of −C215, two fragments covering 455 to 655 a.a. (Frag. A) and 0.5 kb of 3′ UTR (Frag. B) were amplified by two pairs of primers: −C215-F/−C215-R and −C215-3UTR-F/−C215-3UTR-R. These two fragments were serially inserted as a donor into *AflII*/*HindIII* of pBS-1, which was a CRISPR/Cas9 plasmid customized to *P.* *berghei* [39]. For the −C495 mutant, two fragments covering 189–375 a.a. (Frag. C) and 0.5 kb of 3′ UTR (Frag. D) were amplified by two pairs of primers: −C495-F/−C495-R and −C495-3UTR-F/−C495-3UTR-R. These two fragments were inserted into pBS-1, as described above. For the −ZF1 mutant, fragments covering the 5′ UTR (0.5 kb) to the first 10 a.a. (Frag. E) and 38 to 220 a.a. motif (Frag. F) were amplified using −ZF1-F1/−ZF1-R1 and −ZF1-F2/−ZF1-R2. For the −ZF2 mutant construct, fragments covering the 5′ UTR (0.2 kb) to the first 79 a.a. (Frag. G), and 115 a.a. to 270 a.a. (Frag. H) were amplified using −ZF2-F1/−ZF2-R1 and −ZF2-F2/−ZF2-R2, respectively. The fragment, E/F, and G/H were inserted as donors into pBS-1. The PAM sites were searched using CHOPCHOP (http://chopchop.cbu.uib.no, accessed on 12 December 2018). The double-stranded DNA coding for gRNA was inserted into the *BsmBI* site in pBS-1. The resultant plasmids were introduced into *P. berghei*, and transformants were cloned. All clones were subjected to Sanger sequencing to confirm that the expected regions in the *Pb103* gene were deleted. The sequences of primers and gRNAs are listed in Supplementary Table S1.

### 3.9. Statistical Analyses

An unpaired *t*-test or one-way ANOVA with Tukey’s multiple comparison test of means was used to compare the ookinete conversion rate. The Mann–Whitney U-test was used to analyze oocyst density (oocyst number/midgut). Statistical analyses were carried out using GraphPad Prism 9 (GraphPad Software Inc., San Diego, CA, USA, version 9.3.0).

## Figures and Tables

**Table 1:** Gametocytemia and sex ratio of *Pb103(−)* and wild-type.

	Gametocytemia (%)	Sex Ratio (♂:♀)
*Pb103(* *−* *)*	0.24	1:2.38
WT	0.25	1:1.96

The numbers of male and female gametocytes per 10,000 RBC were counted (*n* = 3).

**Table 2:** Infectivity of salivary gland extract from the mosquitoes infected with *Pb103(−)* or wild-type.

Parasites	No. of Salivary Glands	Infected/Injected
WT	5	1/1
	6	1/1
	4	1/1
*Pb103(−)*	15	0/1
	20	0/1
	15	0/1

Mice were intravenously injected with the pooled salivary gland extract from mosquitoes being infected with *Pb103(−)* or wild-type. The blood smear slides were made on day 5 and 16 after injection.

## Data Availability

Not applicable.

## Supplementary Materials

The following are available online at https://www.mdpi.com/article/10.3390/pathogens10121536/s1, Table S1: List of oligos used in this study.
